# Engineered Nanomaterials Suppress the Soft Rot Disease (*Rhizopus stolonifer*) and Slow Down the Loss of Nutrient in Sweet Potato

**DOI:** 10.3390/nano11102572

**Published:** 2021-09-30

**Authors:** Lin-Jiang Pang, Muhammed Adeel, Noman Shakoor, Ke-Rui Guo, Dai-Fu Ma, Muhammad Arslan Ahmad, Guo-Quan Lu, Mei-Hui Zhao, Sheng-E Li, Yu-Kui Rui

**Affiliations:** 1 College of Food and Health, Zhejiang A&F University, Hangzhou 311300, China; ljpang@zafu.edu.cn (L.-J.P.); echo899@163.com (M.-H.Z.); Shengeli31417@126.com (S.-E.L.); 2 School of Life Science, Jiangsu Normal University, Xuzhou 221116, China; 3 Beijing Key Laboratory of Farmland Soil Pollution Prevention and Remediation, College of Resources and Environmental Sciences, China Agricultural University, Beijing 100193, China; Chadeel969@gmail.com (M.A.); lb20203030039@cau.edu.cn (N.S.); guokerui_utokyo@163.com (K.-R.G.); ruiyukui@163.com (Y.-K.R.); 4 BNU-HKUST Laboratory of Green Innovation, Advanced Institute of Natural Sciences, Beijing Normal University Zhuhai Subcampus, 18 Jinfeng Road, Tangjiawan, Zhuhai 519085, China; 5 Laboratory of Soil Science, Graduate School of Agricultural and Life Sciences, The University of Tokyo, Tokyo 113-8657, Japan; 6 Key Laboratory of Biology and Genetic Improvement of Sweet Potato, Ministry of Agriculture and Rural Affairs, Xuzhou Institute of Agricultural Sciences of the Xuhuai District of Jiangsu Province, Xuzhou 221121, China; 7 Shenzhen Key Laboratory of Marine Bioresource and Eco-Environmental Science, College of Life Sciences and Oceanography, Shenzhen University, Shenzhen 518060, China; arslan.slu@gmail.com

**Keywords:** sweet potato, C_60_ nanomaterial, TiO_2_ nanomaterial, CuO nanomaterial, antifungal effect, physiology

## Abstract

About 45% of the world’s fruit and vegetables are wasted, resulting in postharvest losses and contributing to economic losses ranging from $10 billion to $100 billion worldwide. Soft rot disease caused by *Rhizopus stolonifer* leads to postharvest storage losses of sweet potatoes. Nanoscience stands as a new tool in our arsenal against these mounting challenges that will restrict efforts to achieve and maintain global food security. In this study, three nanomaterials (NMs) namely C_60_, CuO, and TiO_2_ were evaluated for their potential application in the restriction of Rhizopus soft rot disease in two cultivars of sweet potato (Y25, J26). CuO NM exhibited a better antifungal effect than C_60_ and TiO_2_ NMs. The contents of three important hormones, indolepropionic acid (IPA), gibberellic acid 3 (GA-3), and indole-3-acetic acid (IAA) in the infected J26 sweet potato treated with 50 mg/L CuO NM were significantly higher than those of the control by 14.5%, 10.8%, and 24.1%. CuO and C_60_ NMs promoted antioxidants in both cultivars of sweet potato. Overall, CuO NM at 50 mg/L exhibited the best antifungal properties, followed by TiO_2_ NM and C_60_ NM, and these results were further confirmed through scanning electron microscope (SEM) analysis. The use of CuO NMs as an antifungal agent in the prevention of *Rhizopus stolonifer* infections in sweet potatoes could greatly reduce postharvest storage and delivery losses.

## 1. Introduction

The consumption of fruits and vegetables has significantly increased in recent years due to the exponential growth of the population. It has been estimated that population growth by 2050 will be 9.8 billion and will require a 70% increase in food production to overcome food security [1]. The postharvest loss poses a serious threat to global food security due to devastating effects on vegetables and fruits during storage. According to a report of Food and Agriculture Organization of the United Nations (FAO) in 2015, about 45% of the world’s fruit and vegetables were wasted, resulting in economic losses ranging from $10 billion to $100 billion worldwide [2]. It has been documented that in developed countries, the loss rate of fresh fruit and vegetables is 20–25% [3,4]. However, developing countries' situation is more severe due to inadequate handling facilities and losses reaching ~50% [5].

The postharvest diseases in crops and fruit causing major losses are primarily controlled using fungicides [6,7]. However, in recent years, the resistance of *Rhizopus stolonifer* to conventional synthetic fungicides [8] has drastically increased because the fact that the widespread, long-term agricultural use of synthetic fungicides has caused some major postharvest pathogens to develop resistance [9]. Furthermore, fungicides have been applied to overcome aforementioned losses and 70–99% of these never reach their target, except for several fungicides [10,11,12]. The presence of these fungicides causes a detrimental impact on soil health and surface water quality as well as by terrestrial organisms [13,14]. Recently, extensive research has been conducted to minimize the heavy dependence on synthetic fungicides for controlling postharvest diseases [15]. However, this mounting challenge needs safe and environmentally friendly solutions for perishable crops including sweet potatoes [15].

Nanoscience stands as a new tool in our arsenal against these mounting challenges that will restrict efforts to achieve and maintain global food security. Previously, studies reported that nanomaterials (NMs) increase the shelf life of fruits as compared to other traditional methods that are used to minimize postharvest losses [16]. For example, studies reported that silicon dioxide [17], titanium dioxide [18], zinc oxide [19], and chitosan [8] NMs are often used in fruit and vegetable preservation as antibacterial and antifungal agents. Adeel et al. reported inhibitory effects of chitosan on black rot disease caused by C. *fimbriata* in sweet potato roots [20] and suggested that Aureofaciens SPS-41 might constitute an attractive biological fumigant for controlling black rot disease in sweet potato roots [21]. Similarly, another study has shown that metal-based NMs have a significant antifungal effect on Botrytis cinerea on rose petals [22]. Interestingly, foliar application of engineered nanomaterials (ENMs) such as Fe_2_O_3_, TiO_2_, MWCNTs, and C_60_ NMs, has been shown to significantly increase biomass and inhibit virus proliferation in tobacco (Nicotiana benthamiana) [23]. Recently, Adeel et al. explored the protective role of carbon-based NMs, with suppression of tobacco mosaic virus (TMV) symptoms via hindered physical movement and viral replication [24]. Farooq et al. described the emerging field challenges and research gaps that are instrumental to the successful development of a nanotechnology-based, multidisciplinary approach for prevention of viral diseases [25].

Sweet potatoes are the seventh largest food crop in the world and the fourth most important in China [26,27]. Due to 70% water content, sweet potato can wilt easily, become discolored, and start decaying, which causes an economic loss of more than 30% [28]. During storage and transportation, sweet potatoes are susceptible to pathogen invasion [29], leading to soft rot, black spot, and other types of disease. Soft rot is caused mainly by *Rhizopus stolonifer*, known as black bread mold, and is a major cause of the loss of sweet potatoes during postharvest storage, with the fungal plant pathogen, Fusarium circinatum, also causing losses [30]. *Rhizopus stolonifer* is widely distributed in the air and soil, so it can infect sweet potatoes, rapidly developing from water stains, to rot the roots completely. The whole rotting process can be completed in just a few days [31]. These pathogens are wound parasites, so they usually require a wound in the skin or stem of the plant to contact susceptible tissue and initiate the infection. The best way to control Rhizopus soft rot is to heal any wounds incurred during harvest and by avoiding any damage during storage. However, injuries occur unavoidably during washing, packing, shipping, and marketing, so these wounds could be a major factor in sporadic outbreaks of Rhizopus soft rot [32].

The application of nanotech for the storage of fruit and vegetables is still in its initial stages, so further investigations, new discoveries, and research breakthroughs are required, especially, for the storage of sweet potatoes. At the same time, new alternatives and less harmful strategies need to be established for controlling and treating infectious diseases caused by phytopathogens. Therefore, the objectives of the present study are to investigate the postharvest application of nanomaterials as a novel strategy for controlling postharvest diseases in sweet potatoes. This study aims to evaluate the antifungal activity of CuO, TiO_2_, and C_60_ NMs against *Rhizopus stolonifer* in sweet potatoes and to determine the effects of nanomaterials on the defense-related activity of several enzymes and hormones and the structure of sweet potatoes. To our knowledge, this is the first study that evaluates the mechanistic physiological, and biochemical, evidence on the impact of different levels of ENMs on fungus inhibition.

## 2. Materials and Methods

### 2.1. Experimental Materials

Two sweet potato cultivars, Y25 and J26, bred by the Yantai Academy of Agricultural Sciences and the Crop Research Institute of Shandong Academy of Agricultural Sciences respectively, were selected for investigating the antifungal effects of selected NMs. The sweet potatoes used in the experiment were provided by the Institute of Dry Farming, Zhanjiang Academy of Agricultural Sciences (Zhanjiang, China). *Rhizopus stolonifer* (*Ehr. ex Fr.*) Vuill was provided by Xuzhou Institute of Agricultural Sciences of the Xuhuai District of Jiangsu Province of China.

### 2.2. Nanomaterial Characterization

The C_60_, CuO, and TiO_2_ NMs, selected as antifungal material for this experiment, were purchased from the Shanghai Pantian Powder Materials Co., Ltd. (Shanghai, China). A transmission electron microscope (TEM) (JEM-2100, JEOL, Tokyo, Japan) was used to determine the shape and size of the NMs before their use in experiments. To prepare the samples for TEM, the NMs were dissolved, sonicated in ethanol, then dropped onto Cu grids. The powders were dispersed in deionized water for observation of zeta potential and dynamic light scattering (DLS).

### 2.3. Preparation of Sweet Potato Inoculant and Inoculation

A small amount of sterile water was added to *Rhizopus stolonifer* (*Ehr. ex Fr.*) Vuill after 7 d of culture, which was then gently scraped with a sterile inoculation ring, filtered through four layers of wipe paper, counted using a hemocytometer, diluted with sterile water, and formulated into a spore suspension at 1 × 10^6^ CFU/mL, ready for use.

The sweet potatoes were washed with tap water then dried in the air. After wiping with a 75% ethanol solution they were placed on a sterile bench. Each variety of sweet potato was deliberately damaged by making two small holes (diameter, 5 mm; depth, 5 mm), 30 mm apart using a sterilized punch. Using a disposable syringe, 25 μL of the bacterial suspension were placed in the holes, allowed to dry, then stored in an artificial climate box (SAFE, Ningbo, China) at a temperature of 28 °C and relative humidity of 85%.

### 2.4. Treatment Groups

We used three NMs at concentrations of 50 and 200 mg/L against two cultivars of sweet potato with their respective controls, eight treatments in total. Each treatment group comprised three replicates, with three parallel samples for each replicate. Each sweet potato inoculated was sprayed with 0.5 mL/cm^2^ NMs solution on the surface and dried out naturally in the air.

### 2.5. SEM (Scanning Electron Microscope) Assessment

A longitudinal cut was made along the infected part of the sweet potato, and a tissue sample (about 1 cm^2^) was taken from the hole previously made. The sample was stored in 2.5% glutaraldehyde fixing solution then dried under vacuum to remove the water before observation by SEM.

### 2.6. Determination of Hormonal Contents of Sweet Potato

The contents of indole-3-acetic acid (IAA), Indolepropionic acid (IPA), brassinolide (BR), gibberellic acid 3 (GA-3), gibberellic acid 4 (GA-4), zeatin riboside (ZR), dihydrozeatin riboside (DHZR), and methyl jasmonate (JA-ME) were determined by following Adeel et al. [23,24]. Those hormonal contents of sweet potato roots were extracted, purified, and quantified using enzyme linked immunosorbent assay (ELISA) after the exposure test [33].

### 2.7. Determination of Enzyme Activity of Sweet Potato

Different enzyme-linked immunosorbent assay (ELISA) kits (Nanjing Jiancheng Bioengineering Institute, Nanjing, China) were used to determine the antioxidant enzyme activity and malondialdehyde (MDA) content using established experimental methods and operations [34,35].

### 2.8. Biochemical Analysis

Frozen sweet potato (2 g) powder obtained by lyophilizing and crushing was repeatedly extracted with 5 mL of ethyl acetate until the extract was colorless. All the extracts were transferred to a 50 mL volumetric flask for a constant volume, then centrifuged at 11,000× *g* for 4 min. The absorbance of the supernatant was evaluated using an ultraviolet/visible spectrophotometer (UV-2802, Unico (Shanghai) Instrument Co., Shanghai, China) at 454 nm. The carotenoids content (g/(100 g)) was calculated as OD_454 nm_ × 10 [36].

#### 2.8.1. Total Flavone Content

To prepare the standard curve, a 0.021 g portion of rutin standard dried at 105 °C was made up to 100 mL with a 30% ethanol solution. Then 0, 0.5, 1.0, 1.5, 2.0, 2.5, and 3.0 mL of this rutin standard solution were each placed in a test tube, made up to 5 mL with 30% ethanol, mixed well, then 0.3 mL of 5% sodium nitrite solution were added. The tubes were shaken well, then allowed to stand for 6 min. After adding 0.3 mL of 10% aluminum nitrate solution, the tubes were mixed for 5 min. Four mL of 1 mol/L sodium hydroxide solution were added, and then 0.4 mL of 30% ethanol to make the total volume up to 10 mL, then shaken well. After standing for 10 min, the absorbance was measured at 510 nm.

#### 2.8.2. Total Phenol Content

It was measured by preparing 0.5 g sample of frozen sweet potato powder added to 10 mL of 60% ethanol solution, ultrasonically extracted for 30 min, centrifuged at 10,000× *g* for 10 min, and then 1 mL of the supernatant was taken. The rest of the procedure was the same as that for the standard method above. The total flavone content (g/(100 g)) was calculated as the weight of rutin (mg) divided by the fresh weight of sweet potato (g). The total phenol content was determined using the Folin–Ciocalteu method. A 0.04 g portion of gallic acid was accurately weighed then dissolved in 10 mL of 95% ethanol and made up to 100 mL with water, to provide a 400 mg/L gallic acid standard solution. Then 1, 2, 3, 4, 5, 6, and 10 mL of this solution were accurately measured using volumetric flasks, to provide the 40, 80, 120, 160, 200, 240, and 400 mg/L gallic acid standard solutions. Then 0.5 mL of the above solution was measured into a 25 mL volumetric flask, mixed with 1 mL of a 1 mol/L solution of Folin phenol reagent, allowed to stand for 5 min, and then 5 mL of 7.5% sodium carbonate solution were added, and made up to 25 mL. After shaking well, the solutions were heated in a water bath at 40 °C for 30 min. The absorbance was then adjusted to zero using distilled water, and the solution absorbance was measured at 765 nm. The standard curve was used to obtain the final result.

For sample measurement, 2 g of sweet potato were accurately weighed then added to 20 mL of 80% methanol in a centrifuge tube, shaken, sonicated at 40 °C for 30 min in the dark, and centrifuged at 4 °C and 9000× *g* for 30 min. A 0.5 mL sample of the sweet potato extract was measured following the same procedure as for preparing the standard curve. The total phenol content (g/(100 g)) was calculated as the weight of gallic acid (mg) divided by the fresh weight of sweet potato (g).

#### 2.8.3. Ascorbic Acid Content

It was measured by preparing the standard curve; 1.5 g of activated carbon was added to 50 mL of standard solution (Vitamin C, 1 mg/mL), shaken well for full oxidation, then filtered. Ten milliliters of the filtrate was placed in a 500 mL volumetric flask, 5.0 g of thiourea was added, and then made up to 500 mL, with 1% oxalic acid solution for later use. Then 5, 10, 20, 25, 40, 50, and 60 mL of this diluted solution were placed into 100 mL volumetric flasks and made up to 100 mL with 1% thiourea solution. Using this sample measurement procedure, tritium is formed so that the colors can be compared. The standard curve was drawn with absorbance as the ordinate and ascorbic acid concentration (μg/mL) as the abscissa.

### 2.9. Data Analysis

The results were expressed as the mean ± standard deviation from triplicate samples. All the data were analyzed by one-way analysis of variance with Duncan’s test using the SPSS 19.0 statistical software package for Windows (IBM Corp., Armonk, NY, USA). Differences were considered statistically significant at *p* < 0.05.

## 3. Results and Discussion

This section is divided by subheadings and provides a concise and precise description of the experimental results, their interpretation, as well as the experimental conclusions that can be drawn.

### 3.1. NMs Characterization

**Figure 1:**
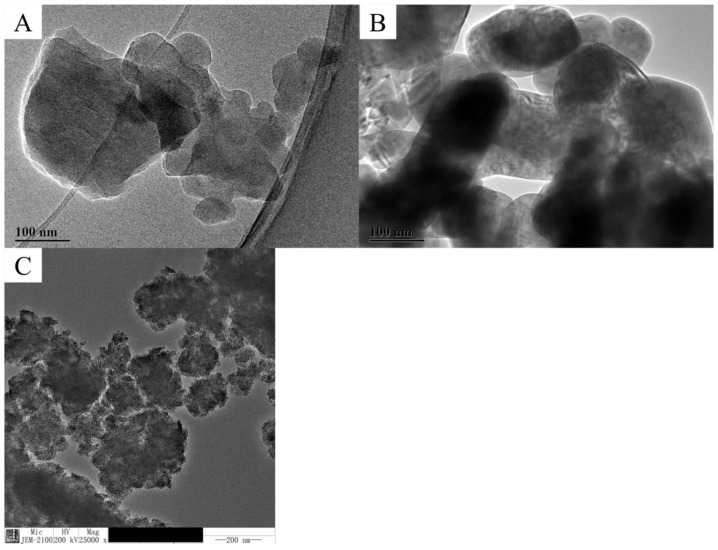
Transmission electron microscopic (TEM) images of (**A**): Carbon 60 NMs; (**B**): Copper oxide NMs and (**C**): Titanium dioxide NMs.

The average diameter of the carbon 60 (C_60_) NM was approximately around 70 nm (Figure 1A) with that of the copper oxide (CuO) NM ranging from 70 to 80 nm (Figure 1B) and that of the titanium dioxide (TiO_2_) NM approximately 75 nm (Figure 1C). The zeta (ζ) potential of the C_60_ NM dispersed in deionized water at 100 mg/L was −18.933 ± 1.501 mV, 943.100 ± 6.788 nm; that of the CuO NM dispersed in water at 100 mg/L was 14.900 ± 1.217 mV, 2790.000 ± 19.799 nm; and that of the TiO_2_ NM dispersed in water at 0.5 mg/mL was −12.967 ± 0.950 mV, 1110.000 ± 79.196 nm. Results of Zeta potential show moderate stability of all applied nanomaterials.

### 3.2. NMs Treatment Suppressed the Soft Rot Symptoms and Disease Development

**Figure 2:**
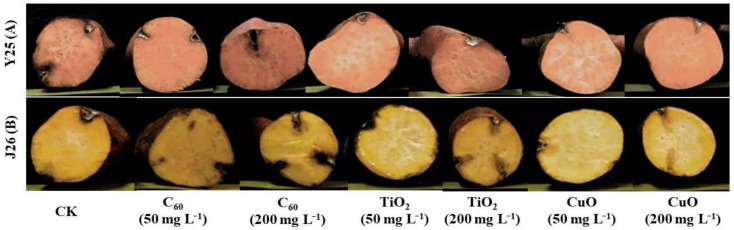
Representative images of the cross sections of sweet potatoes inoculated against soft rot: (**A**) representing Y25 cultivar and (**B**) representing J26 cultivar of sweet potato at different concentrations of NMs. CK is not sprayed with any NMs.

The potential role of NMs in promoting the plant post-harvesting overall quality and biotic/abiotic stress tolerance has been well documented [37,38]. Recently, it has been reposted that several NMs act as anti-pathogenic agents and play vital roles in disease prevention [24,39]. The transverse sections of the Y25 cultivar of sweet potatoes (Figure 2A) showed that the plaque depth after CuO NM treatment was very shallow, with the degree of soft rot being less than those of the other treatment groups. However, sweet potatoes from the C_60_ NM treatment group exhibited serious soft rot.

The transverse sections of the J26 cultivar of sweet potatoes (Figure 2B) showed that the plaque depth and infection area of the C_60_ NM treatment group were significantly greater than those of the other treatment groups. Regarding the degree of soft rot, the softened condition of sweet potatoes in the C_60_ NM treatment group was more serious with an obvious alcohol aroma, but the TiO_2_ NM and CuO NM treatments exhibited good antifungal effects. Our findings are in agreement with previous studies, as early studies also reported that metal-based NPs are used as a fungicide against the soft rot of potatoes [40]. Nafady et al. reported the fungicidal impacts of ZnO NPs against soft rot of sweet potato at 50 ppm applied concentration. Whereas in the current study CuO NMs produces a significant antifungal effect as compared to other applied materials. The application of three different types of NMs in this study is the novel aspect of this work as previously published literature focused on one type of NM [41].

**Figure 3:**
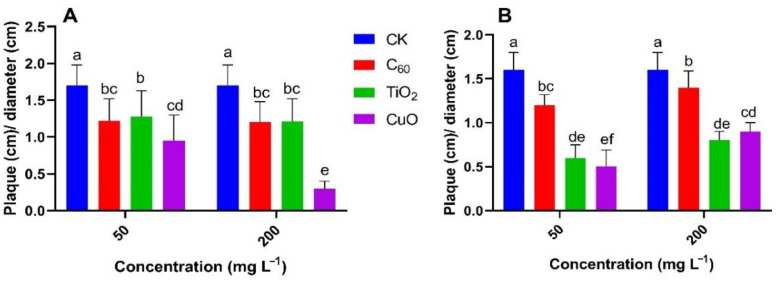
Effect of nanomaterial treatment on the mean ratio of plaque depth (cm) to plaque diameter (cm) for two sweet potato cultivars: (**A**) Y25 and (**B**) J26. Different small letters represent significant difference according to Duncan’s multiple range test (*p* < 0.05. n = 3).

**Figure 4:**
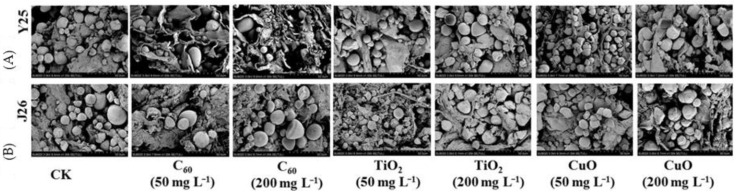
Representative scanning electron microscopic (SEM) images of the cross sections of inoculated sweet potato (**A**) representing Y25 cultivar and (**B**) representing J26 cultivar of sweet potato at different concentrations of NMs.

For both the Y25 and J26 sweet potato cultivars, we measured plaque depth (cm)/diameter (cm) as shown in Figure 3 and the CuO treatment provided the best level of fungistasis followed by the TiO_2_ NM and C_60_ NM treatments. Our results were further confirmed by scanning electron microscope (SEM) images (Figure 4).

The SEM images (Figure 4A,B) showed that the antifungal effect on Y25 sweet potatoes was better than that on J26 sweet potatoes. Compared with the control, the tissue rot at the two concentrations of C_60_ NM was poorly contained. The CuO NM treatment exhibited a good antifungal effect, with tissue rot at both concentrations being less. For Y25 sweet potatoes, the TiO_2_ NM treatment provided a better antifungal effect with less tissue rot observed compared with the control.

The SEM images showed that the extent of tissue rot on the 200 mg/L C_60_ NM treatment group was slightly improved compared with the control, but the 50 mg/L C_60_ treatment offered no significant improvement. For sweet potatoes treated with CuO NM, the tissue rot improved at both concentrations. The SEM images showed that the two concentrations of TiO_2_ NM did not provide good inhibition of sweet potato rot. In summary, phenotypic observation, SEM imaging, and plaque depth (cm) to plaque diameter (cm) for two sweet potato cultivars clearly demonstrate that CuO NMs at 50 mg/L were highly effective at suppressing the development of soft rot symptoms and disease in sweet potatoes. The exact mechanism behind the excellent fungicidal potential of CuO NMs needs to be explored yet. One possible explanation is the production of Cu^2+^ ions and their effect on the growth and metabolism of *Rhizopus stolonifer*, which leads to inhibition of fungus production [41].

### 3.3. NMs Application Enhances Hormonal Contents of Sweet Potato

Plant hormones regulate many processes during plant growth and development [33], so the effects of nanomaterials on plant hormones should be an important index for judging their antifungal effect. Indole-3-acetic acid (IAA) is the most prominent phytohormone in plants, playing a very important role in the process of plant growth and development. Auxin-like hormones affect growth, maturation, and senescence at the organ level, and, at the cellular level, can affect cell elongation, division, and differentiation [1,2,3,42,43]. Brassinolide (BR) shows some characteristics of auxin and gibberellin, being able to promote vegetative growth, flower bud differentiation, and the growth of plants. BR also mediates plant responses to various abiotic and biotic stresses [44,45]. Gibberellic acid (GA) is an endogenous plant hormone, which can stimulate plant growth and development. GA has been synthesized and generally used as a growth promoter in production by promoting seed germination and the growth of plant stems and leaves, regulating flowering, increasing yield, and improving quality [46,47]. Indolepropionic acid (IPA) also participates in many important metabolic processes in plants [48]. Overall, these four hormones can regulate the growth of plants and enhance their tolerance to abiotic stress.

**Figure 5:**
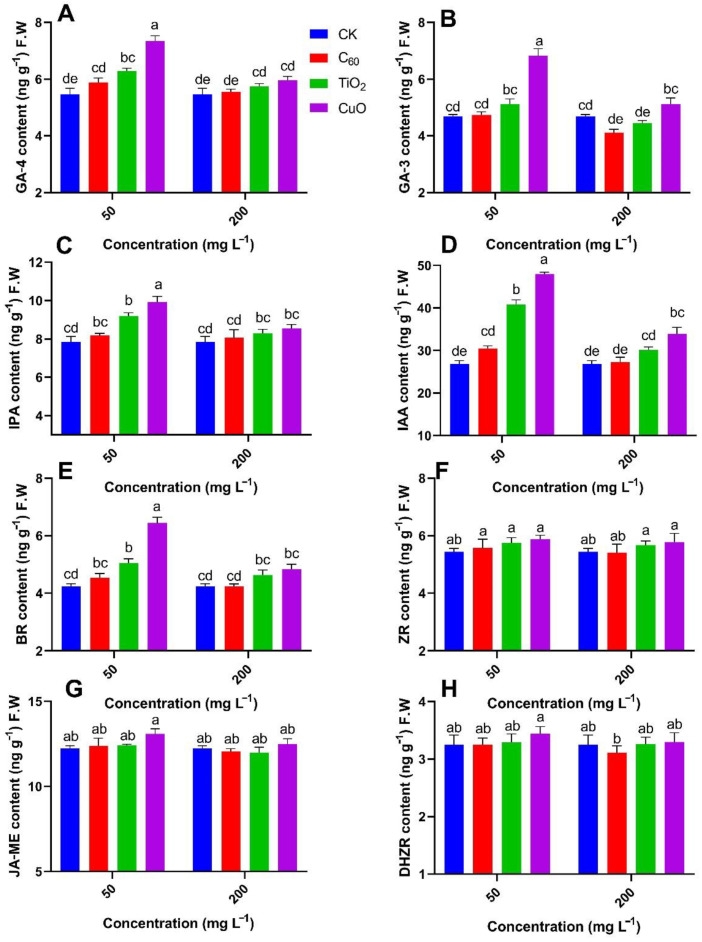
Effect of different NMs on the mean contents (n = 3) of (**A**) gibberellic acid-4, (**B**) gibberellic acid-3, (**C**) indolepropionic acid, (**D**) indole-3-acetic acid, (**E**) brassinolide, (**F**) zeatin riboside, (**G**) methyl jasmonate, and (**H**) dihydrozeatin riboside hormones in sweet potato cultivar (Y25) exposed to soft rot disease. Different small letters represent significant difference according to Duncan’s multiple range test (*p* < 0.05. n = 3).

An analysis of the contents of GA, IPA, BR, and IAA, which play an important role in the regulation of plant growth and development in Y25 sweet potatoes (Figure 5A–E), leads to the following findings.

The content of GA-4 in sweet potatoes from the CuO NMs treatment group was significantly higher (34%) from those of the control group followed by TiO_2_ NMs (15%) group, respectively, at 50 mg/kg of applied concentration. The lowest content GA-4 was observed in the case of C_60_ NMs treatment at 50 mg/kg of applied concentration. A similar trend was noted in GA-3 but the TiO_2_ NMs and CuO NMs treatment did not show the significant result at 50 mg/kg of applied concentration. IPA, IAA, and BR content was significantly increased with the inoculation of CuO NMs of 26%, 78%, and 51%, respectively, at 50 mg/kg of applied concentration as compared to control. With the increase of applied NMs concentration, a downward result was observed for the production of hormonal content.

Overall, analysis of the four hormones (GA, IPA, BR, and IAA) that have a great influence on plant growth and development showed that the TiO_2_ NMs treatment provided the highest antifungal effect, followed by C_60_ NMs and CuO NMs, respectively. The contents of dihydrozeatin riboside (DHZR), zeatin riboside (ZR), and methyl jasmonate (JA-ME) from each treatment group were not significantly different from those of the control group (Figure 5F–H).

**Figure 6:**
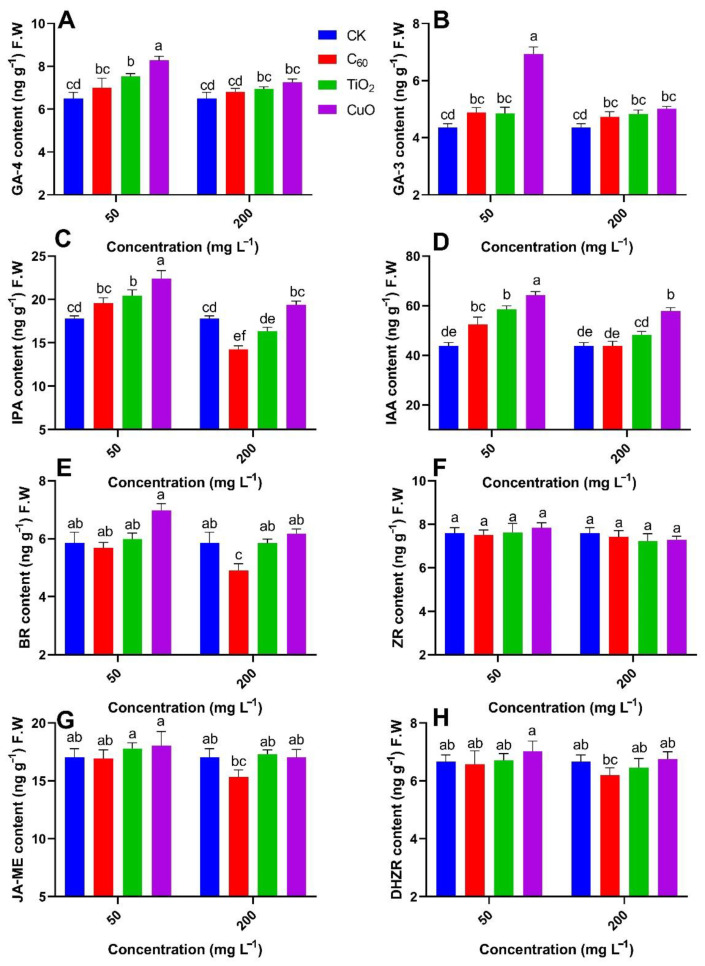
Effect of different NMs on the mean contents (n = 3) of (**A**) gibberellic acid-4, (**B**) gibberellic acid-3, (**C**) indolepropionic acid, (**D**) indole-3-acetic acid, (**E**) brassinolide, (**F**) zeatin riboside, (**G**) methyl jasmonate, and (**H**) dihydrozeatin riboside hormones in sweet potato cultivar (J26) exposed to soft rot disease. Different small letters represent significant difference according to Duncan’s multiple range test (*p* < 0.05. n = 3).

Our results lead to the following findings for the contents of GA, IPA, BR, and IAA in J26 cultivars of sweet potato (Figure 6A–E). The content of GA-4 in sweet potatoes from the CuO NMs treatment group was significantly higher (27%) than those of the control group followed by TiO_2_ NMs (15%) group, respectively, at 50 mg/kg of applied concentration. The lowest content GA-4 was observed in the case of C_60_ NMs (7%) treatment at 50 mg/kg of applied concentration. A similar trend was noted in GA-3 where CuO NMs treatment group was significantly higher (59%) and interestingly TiO_2_ NMs and C_60_ NMs show similar trends and reported (11%) of GA-3 content. IPA, IAA, and BR contents were significantly increased with the inoculation of CuO NMs of 25%, 46%, and 19%, respectively, at 50 mg/kg of applied concentration as compared to control. However, C_60_ significantly shows the downward result for hormone production for IPA, BR, and IAA with −19%, −16%, and −0.25%, respectively, at 200 mg/kg of applied concentrations.

Overall, analysis of the four hormones including GA, IPA, BR, and IAA showed that the TiO_2_ NMs treatment provided the lowest antifungal effect, followed by C_60_ NMs, with CuO NMs providing the best effect at 50 mg/kg of applied concentration as compared to control. The contents of dihydrozeatin riboside (DHZR), zeatin riboside (ZR), and methyl jasmonate (JA-ME) from each treatment group were not significantly different from those of the control group (Figure 6F–H). Conclusively, we observed better hormonal content in Y25 cultivar of sweet potato as compared to J26 cultivar. The CuO NM treatment shows the best antifungal effect followed by the TiO_2_ NM treatment group, with the worst being the C_60_ NM treatment in both cultivars at 50 mg/kg of applied concentration as compared to control. Shang et al. observed that the use of CuO NP partially helps the plant to balance the water in shoots, and thus controls ABA (abscisic acid) production in the plant tissues, consequently stimulating the plant response against abiotic stress [49]. Our findings suggest that CuO NP enabled crosstalk between different hormonal contents in sweet potato exposed to soft rot disease and effectively enhanced the defense response, thus promoting the freshness of sweet potato. Increase in concentration of applied nanomaterial decreases the antifungal effect in all treatments

### 3.4. Activation of the Antioxidant Activities in Sweet Potato over Application of NMs

**Figure 7:**
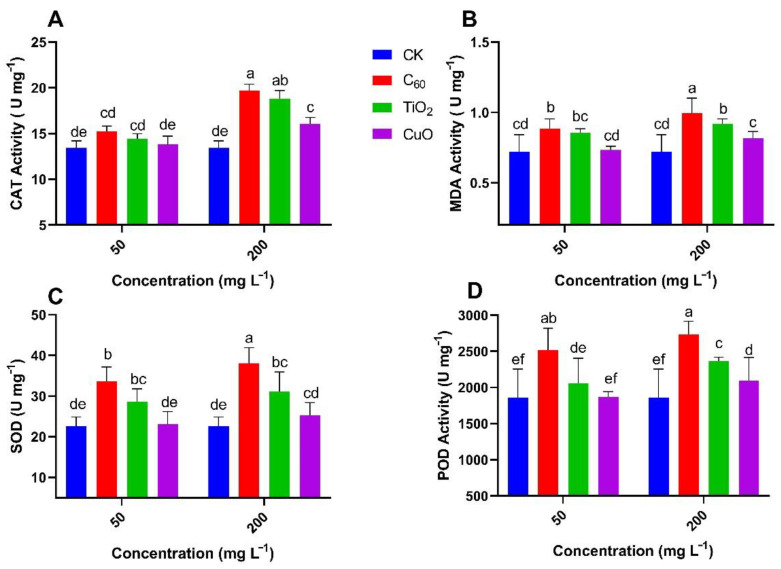
Effect of NMs treatment on the mean content (n = 3) of (**A**) catalase, (**B**) malondialdehyde, (**C**) superoxide dismutase, and (**D**) peroxidase enzymes in sweet potato (Y25) exposed to soft rot disease. Different small letters represent significant difference according to Duncan’s multiple range test (*p* < 0.05. n = 3).

Reactive oxygen species (ROS) induce defense mechanisms against abiotic stress and activate antioxidant systems [50]. Little research has been conducted to elaborate the combined effects of NMs and soft rot disease in sweet potatoes on the production of antioxidant enzymes. ROS enzyme activity including CAT (catalase), MDA, SOD (superoxide dismutase), and POD (peroxidase) was measured in NM-treated sweet potatoes that were infected with soft rot (Figure 7A–D).

Antioxidant enzymes play an important role in detoxifying sources of abiotic stress [51,52]. Stress can increase the production of reactive oxygen species (ROS), which destroy biological macromolecules and reduce enzyme activity. Reducing stress can thus help to increase the content of some protective enzymes such as peroxidase (POD) and catalase (CAT) [53,54]. In plants, CAT mainly removes hydrogen peroxide produced during electron transport, β-fatty acid oxidation, and photorespiration, thus preventing damage caused by ROS free radicals to plants [55]. CAT also plays an important role in plant growth and development, stress defense response, oxidative senescence, and other physiological processes. Its expression activity is also affected by many biological and abiotic factors, such as light, temperature, high salt, drought, plant hormones, and other pathogenic microorganisms [56]. As a protective enzyme of the in vivo defense system, POD can effectively catalyze the decomposition of hydrogen peroxide into water, thus effectively preventing its accumulation in vivo and eliminating potential damage to the cell membrane structure of leaves [57].

Superoxide dismutase (SOD) is the first line of defense against an active oxygen-scavenging system, which can convert O^-^ into O_2_ and H_2_O_2_, thus reducing the toxic effect of ROS free radicals on cells [58,59]. Malondialdehyde (MDA) is the final decomposition product of membrane lipid peroxide, so it indicates the degree of membrane oxidation in plant cells [56].

The enzyme activity determined in Y25 cultivar of sweet potatoes (Figure 7) showed that the activities of CAT, MDA, SOD, and POD at the 50 mg/L of CuO NMs group was non-significant (0–2%) as compared to control. With C_60_ NMs treatment, the activities of CAT, MDA, SOD, and POD at the 50 mg/L were significantly higher (30–49%) as compared to control. TiO_2_ NMs treatment group showed a significant increase for MDA (18%) and SOD (26%), respectively, and insignificant for CAT (7%) and POD (10%) as compared to the control group at 50 mg/L of applied concentration. All enzymatic activites were increased with the increase of applied NMs concentration but the lowest increase was observed for the CuO NMs group.

**Figure 8:**
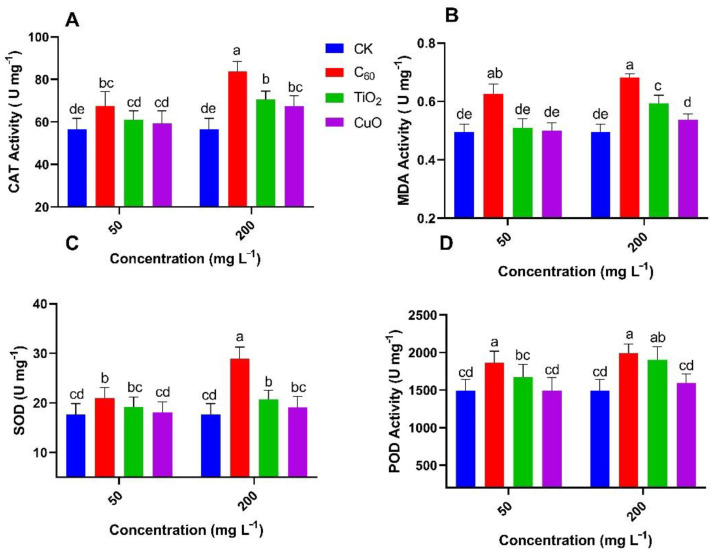
Effect of NMs treatment on the mean content (n = 3) of (**A**) catalase, (**B**) malondialdehyde, (**C**) superoxide dismutase and (**D**) peroxidase enzymes in sweet potato (J26) exposed to soft rot disease. Different small letters represent significant difference according to Duncan’s multiple range test (*p* < 0.05. n = 3).

The enzymatic activities observed in J26 cultivar of sweet potatoes (Figure 8) showed that the activities of CAT, MDA, SOD, and POD at the 50 mg/L of CuO NMs group was non-significant (0–4%) as compared to control. TiO_2_ NMs treatment group showed a significant increase for POD (12%) and a non-significant increase in the case of MDA (2%), CAT (7.9%), and POD (7.7%) as compared to the control group at 50 mg/L of applied concentration. With C_60_ NMs treatment, the activities of CAT, MDA, SOD, and POD at the 50 mg/L was significantly higher (19–25%) as compared to control. All enzymatic activities were increased with the increase of applied NMs concentration but the lowest increase was observed for the CuO NMs group. However, modulation in enzymatic activity for J26 was not as significant as the Y25 cultivar of sweet potato.

The antimicrobial/fungal pathogens of the applied nanomaterials might be attributed to accumulate ROS, which caused oxidative stress in cells, including lipid peroxidation in the cell membrane leading to leakage of intracellular contents, DNA damage, and inhibition of partial enzyme activities connecting with cell growth; interacted with respiratory chain enzymes, leading to disruption of adenosine triphosphate production; and caused protein dysfunction, including destruction of Fe-S clusters, the cellular donor ligands that coordinate Fe and exchange of catalytic or/and structural metal, resulting in the death of phytopathogens [19,60,61]. These findings can guide researchers and other stakeholders dealing with post harvesting of sweet potato regarding the selection of specific nanoparticles for their use as antifungal agents.

### 3.5. Nutrients Content of Sweet Potatoes

Vitamin C (Vc), an indispensable product of plant metabolism, is also involved in plant antioxidants [62], photosynthesis [63,64], and metabolic regulation [65]. Vc can rapidly react with ROS and participate in their removal during aerobic metabolism [66]. Vc can also maintain the reduced state of fat-soluble antioxidants, thereby protecting the body and normal metabolism from oxidative stress [67]. Other studies have shown that Vc is involved in the photosynthetic system and mitochondrial electron transfer [68]. In plant cells, carotenoids are mainly located in plastids and participate in physiological functions such as photomorphogenesis and photoprotection [69]. Some oxolytic products of carotenoids are also important precursors of phytohormones such as abscisic acid (ABA) and strigolactones, so are closely related to the growth and development of higher plants [70]. Phenols are important secondary metabolites of plants, being widely present in plants and participating in many physiological and biochemical reactions. Flavonoids play an important role in plant growth, development, flowering, and fruiting, with antibacterial and disease-prevention properties [71].

**Figure 9:**
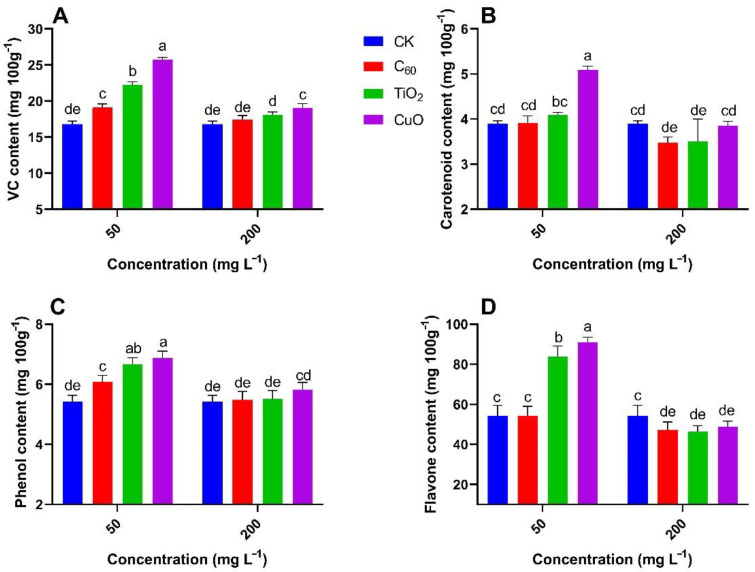
Effect of NMs treatment on the mean content (n = 3) of ascorbic acid (**A**), carotenoids (**B**), total phenol (**C**), and total flavones (**D**) in sweet potato (Y25) exposed to soft rot disease. Different small letters represent significant difference according to Duncan’s multiple range test (*p* < 0.05. n = 3).

**Figure 10:**
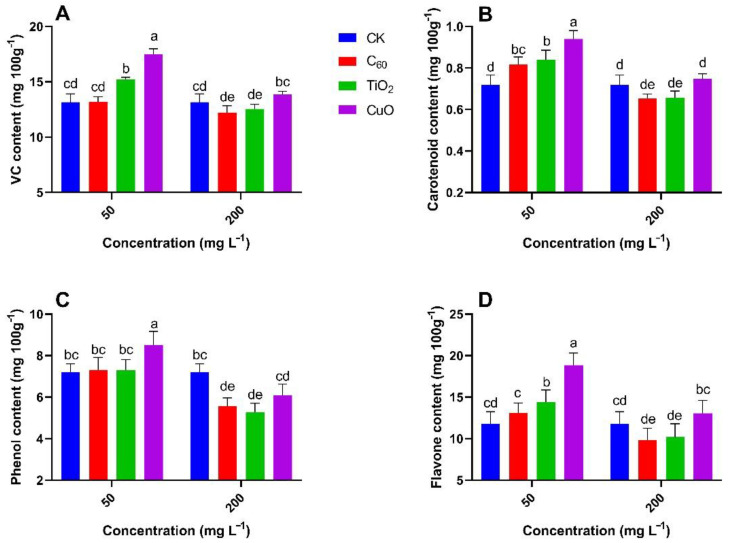
Effect of NMs treatment on the mean content (n = 3) of ascorbic acid (**A**), carotenoids (**B**), total phenol (**C**), and total flavones (**D**) in sweet potato (J26) exposed to soft rot disease. Different small letters represent significant difference according to Duncan’s multiple range test (*p* < 0.05. n = 3).

The antifungal effect of the NMs and the feasibility of their practical use can be measured by analyzing changes in the contents of the main nutrients in sweet potatoes. Figure 9 and Figure 10 show that the contents of nutrients at the 50 mg/L CuO NMs treatment group were significantly higher (26–76%) as compared to the control group, with CuO NMs still exhibiting good antifungal properties. Overall, the nutrient content from the TiO_2_ NMs treatment was not significantly (5–54%) different from that of the control group, indicating that although TiO_2_ NMs could inhibit the development of soft rot, it would not be the most suitable antifungal treatment for sweet potato transport and storage. The C_60_ NMs treatment did not produce good results at both concentrations and was not significantly (0.5–13%) different from the control group. CuO NMs treatment enhances the nutrient content of sweet potato including Vc, carotenoid, phenol, and flavone content at 50 mg/L of applied concentration. Shang et al. observed the increase in nutrient supply and growth of *Lactuca sativa* exposed to *Fusarium oxysporum f. sp*. *Lactucae*, CuO NMs embedded with hydrogels increase the uptake of P, Zn, Mn, and Mg by increasing the levels of organic acids as compared to the *Fusarium oxysporum f. sp* exposed control *Lactuca sativa* [49]. Altogether, our results provide useful information and a nano approach improving the efficiency of NMs for preventing and controlling soft rot in sweet potatoes during their post harvesting.

## 4. Conclusions

This study has investigated the use of novel engineered nanomaterials for preventing and controlling soft rot in sweet potatoes during their storage and transportation. It has also analyzed the infection of the cut surface of sweet potatoes; the contents of the plant hormones GA-3, IPA, GA-4, ZR, IAA, DHZR, JA-ME, and BR; the activities of the plant stress enzymes CAT, MDA, SOD, and POD; the contents of the main nutrients: ascorbic acid, carotenoids, total phenol, and total flavones; and studied the antifungal effect of each nanomaterial. The results for Y25 sweet potatoes showed that treatment with 50 mg/L CuO NM exhibited the greatest antifungal effect, with hormone and nutrient contents generally higher than those of the control group. This indicated that CuO NM was the most practical antifungal agent. The next most effective antifungal effect was provided by TiO_2_ NM, with the least effective being C_60_ NM. When analyzing the J26 sweet potato, the results showed that CuO NM still provided the best level of fungistasis, followed by TiO_2_ NM and C_60_ NM.
